# Endocannabinoids in Alzheimer's disease and their impact on normative cognitive performance: a case-control and cohort study

**DOI:** 10.1186/1476-511X-8-2

**Published:** 2009-01-14

**Authors:** Jeremy Koppel, Heather Bradshaw, Terry E Goldberg, Houman Khalili, Philippe Marambaud, Michael J Walker, Mauricio Pazos, Marc L Gordon, Erica Christen, Peter Davies

**Affiliations:** 1 The Litwin-Zucker Research Center for the Study of Alzheimer's Disease and Memory Disorders, The Feinstein Institute for Medical Research, Manhasset, NY, 11030, USA; 2 The Kinsey Institute for Research in Sex, Gender and Reproduction, Indiana University, Bloomington, IN 47405, USA; 3 The Center for Genomics and Human Genetics, The Feinstein Institute for Medical Research, Manhasset, NY, 11030, USA; 4 Department of Psychological and Brain Sciences, Indiana University, Bloomington, IN 47405, USA; 5 The Albert Einstein College of Medicine and The Litwin-Zucker Research Center for the Study of Alzheimer's Disease and Memory Disorders, The Feinstein Institute for Medical Research, Manhasset, NY, 11030, USA

## Abstract

**Background:**

Neuropathological, animal, and cell culture studies point to a role for the body's own endogenous cannabinoids (eCBs) system in Alzheimer's disease (AD) pathology and treatment. To date, no published studies have investigated the potential utility of circulating eCBs as diagnostic biomarkers for AD or the impact of central eCBs on cognition.

**Results:**

In comparison with healthy controls, there were no significant differences in measured eCB concentrations in plasma samples from patients with AD. Detectable eCBs in cerebrospinal fluid (CSF) had no relationship to cognitive performance in healthy controls at risk for AD. In pooled plasma samples, an inverse correlation was observed between plasma levels of the eCB 2-AG (2-arachidonoylglycerol) and TNF-α (r = -0.41, p < 0.02).

**Conclusion:**

These results suggest that circulating endocannabinoids do not have utility as diagnostic biomarkers for AD and do not have a robust correlation with cognitive performance. Circulating levels of 2-AG may downregulate TNF-α production.

## Background

The known components of the body's endocannabinoid (eCB) system include at least two biolipids structurally identified as *N*-arachidonoylethanolamide (AEA) and 2-arachidonoylglycerol (2-AG) produced "on-demand" from cell membrane precursors in a variety of cell types including both neurons and immune-competent cells in the periphery (B cells, T cells, monocytes/macrophages) and central nervous system (microglia, astrocytes); degradative enzymes; and at least two cannabinoid receptors (CB_1 _and CB_2_) [1]. Several *in vitro *experiments suggest that eCBs act as immune modulators, generally through exerting control over inflammatory cytokine production via the CB_2 _receptor [2-4]. However, it is not yet clear whether circulating eCBs exert control over the inflammatory system *in vivo*.

Recent *in vitro *and *in vivo *studies implicated the eCB system in Alzheimer's disease (AD) pathophysiology and there is a burgeoning sense of excitement about the potential role for eCB system interventions in disease modification (for a recent review see Koppel 2008) [5-11]. Perturbations in plasma and CSF endocannabinoids have been detected in several psychiatric and neurologic disorders including schizophrenia[12,13], multiple sclerosis[14], depression[15], migraine headaches[16,17], and eating disorders[18]. To date, no published studies have investigated plasma levels of eCBs in AD.

While the effects of administered Δ^9 ^-THC (acting at the neuronal CB_1 _receptor) on verbal learning and memory are well established [19], the relevance of eCBs acting on the same receptor to learning and memory is not clear. Acute and chronic use of marijuana impair immediate recall, short-term memory, and memory retrieval [20]. *In vitro *experiments suggest that eCBs may have a more salutary effect; they promote changes in neural activities related to memory, with a potential role in long-term synaptic plasticity [21]. However, in a stress model of depression in mice, reductions in hippocampal 2-AG levels were associated with deficits in behavioral flexibility (suggested to be related to the inability to forget), implicating hippocampal endocannabinoid signaling in the "pruning" of normative memory systems [22]. To date, no published reports establish the impact of central concentrations of eCBs on human cognition.

In a case-control study we compared circulating levels of plasma eCBs in AD and elderly controls and explored the relationship between plasma eCBs, TNF-α and C-reactive protein in order to determine whether circulating eCBs influence inflammatory biomarkers. Previous experiments in cell culture have demonstrated that synthetic cannabinoids downregulate TNF-α in a concentration dependent manner,[2] and we wondered whether that might also be the case with endogenous cannabinoids *in vivo*. We also wondered whether eCBs might have utility as general markers of inflammation, and for that reason chose to investigate their relationship with C-reactive protein. In a study of a separate cohort of healthy normal controls we examined the impact of central (CSF) levels of eCBs on cognition.

## Methods

### AD subjects and elderly controls

Late-onset (over 65 years) AD subjects and elderly controls (over 65) were recruited from the Litwin-Zucker Research Center for the Study of Alzheimer's Disease (LZRC). The research was approved by the North Shore-LIJ institutional review board. Informed consent was provided by subjects, and where appropriate, health care proxy as determined by the institutional review board. All AD subjects met DSM IV [23] and NINCDS-ADRDA [24] criteria for Alzheimer's disease. Elderly controls (without a subjective cognitive complaint) required a score of 28 or higher on the Folstein MMSE. Cases and controls were excluded if they had history of Schizophrenia, Bipolar disorder, Major Depression, Eating Disorder, autoimmune disease, or malignancy, which could conceivably alter eCB concentrations. In order to isolate plasma and protect from enzymatic degradation of eCBs, in all subjects approximately 40 cc of blood was collected in the morning by venopuncture, spun for 3 minutes within 5 minutes at 7200 RPM to facilitate plasma extraction, and flash frozen at -80°C. Samples were transferred to the Feinstein Biorepository and LZRC labs for DNA extraction and amplification as well as cytokine analysis; the North Shore-Long Island Jewish Core Laboratories for C-reactive protein analysis; and were shipped overnight on dry ice to Indiana University for eCB analysis.

### Cognitive controls

Subjects were recruited from the LZRC as part of a longitudinal study investigating CSF biomarkers in cognition and AD. The research was approaved by the North Shore-LIJ institutional review board. All subjects provided informed consent. The majority of these subjects had a positive family history of AD, but did not have cognitive complaints at the time of examination, and none met criteria for dementia. Each subject in the study was administered the following cognitive tests over two days: Mini Mental State Exam (global cognition), Selective Reminding (total recall), Logical Memory I (episodic memory), Trail Making A and B (speed of processing), N Back (executive functioning), Letter and Semantic Fluency (language production and speed)[25].

Lumbar puncture (LP) was performed on one of the two days of testing.

After routine medical and physical assessments, LP was performed in a sitting or lateral decubitus position. Following sterile preparation and local anesthesia with 4% lidocane topical cream and 1% lidocaine subcutaneous injection, a 4 cm long, 20-gauge cutting-tip needle was used as an introducer in the L3-L4 or L4-L5 interspace. Next, a 25-gauge, Whiteacre-point spinal needle was inserted through the introducer and placed in the thecal sac. After fluid return was established, the spinal catheter was connected to a 5 or 10 mL syringe via a polypropylene tube. Gentle negative pressure sufficient to remove CSF at a rate of approximately 2 mL/min was then applied by syringe and approximately 25–30 mL of clear spinal fluid was removed from each subject. CSF was then frozen and stored at -80°C and shipped overnight on dry ice to Indiana University for eCB analysis.

### Drugs and reagents for eCB quantification

High performance liquid chromatography (HPLC) grade water, methanol, and acetonitrile used for mass spectrometric studies were purchased from VWR international, Plainview, NY. Mass spectrometry/HPLC grade acetic acid, formic acid, and ammonium acetate were purchased from Sigma-Aldrich. [^2^H_8_] *N*-arachidonoyl ethanolamine (D_8_-AEA) was purchased from Cayman Chemical (Ann Arbor, MI).

### Lipid extraction and endocannabinoid quantification methods

Lipid extractions were performed on human plasma with the addition 9.5 milliliters of a 1:1 mixture of methanol and acetonitrile to each 1.5 ml of plasma. Lipid extracts of cerebral spinal fluid (CSF) were made by first adding 9.0 milliliters of a 1:1 mixture of methanol and acetonitrile to 1.0 ml CSF. An internal standard of 50 picomoles of [^2^H_8_] *N*-arachidonoyl ethanolamine was spiked into each sample to measure recovery. This solution was vortexed for 1 minute then underwent centrifugation at 19,000 × g at 24°C for 20 minutes. The supernatant was diluted with HPLC water to reach a concentration of 25% organic. Lipids were partially purified from plasma on C8 solid phase extraction columns from which the final eluent of 100% methanol was further extracted on C18 solid phase extraction columns. Lipids were partially purified from CSF on C18 solid phase extraction columns only. In brief, each 500 mg (plasma) or 100 mg (CSF) column was conditioned with 5.0 ml methanol and 2.5 ml water followed by loading of the water/supernatant solution. Columns were then washed with 2 ml water and 1.5 ml 55% methanol. Compounds were eluted with 1.5 ml 100% methanol. The final elution from the C18 column was used for analysis of anandamide and 2-AG.

Rapid separation of analytes was obtained using 20 μl injections of the 100% methanol elution (Agilent 1100 series autosampler, Wilmington, DE) onto a Zorbax eclipse XDB 2.1 × 50 mm reversed phase column. Gradient elution using mobile phase A of 20% methanol, 1 mM ammonium acetate and 0.5% acetic acid and mobile phase B of 100% methanol, 1 mM ammonium acetate and 0.5% acetic acid (200 μl/min) was formed under pressure on a pair of Shimadzu (Columbia, Maryland) 10AdVP pumps. Mass spectrometric analyses were performed with an Applied Biosystems/MDS Sciex (Foster City, CA) API 3000 triple quadrupole mass spectrometer and an API 4000 QTRAP that were both equipped with an electrospray ionization source. Levels of each compound were analyzed by multiple reactions monitoring (MRM) on the LC/MS/MS systems as previously reported [26]. Mass spectrometric conditions were optimized for each compound using direct flow injection of synthetic standards of each compound.

### TNF-α/C-reactive protein

Plasma *TNF*-α ELISA was performed at the LZRC using R and D Systems Quantikine High Senstivity TNF-α/TNFSF1A Immunoassay for serum and plasma (#HSTAOOD). C-reactive protein was quantified with an immunoturbidimetric assay.

### Statistical analysis

For endocannabinoid quantification, we compared AEA and 2-AG values between AD subjects and controls using a non-paired t test. We correlated AEA and 2-AG values with CRP and TNF-α using Pearson's r. One subject, whose 2-AG levels were over three and a half standard deviations from the mean, was excluded from analysis. For analysis of cognition, we correlated 2-AG values with cognitive test performance using Spearman's rank order method so as to reduce the chance that outliers might "drive" a finding.

## Results and discussion

**Figure 1:**
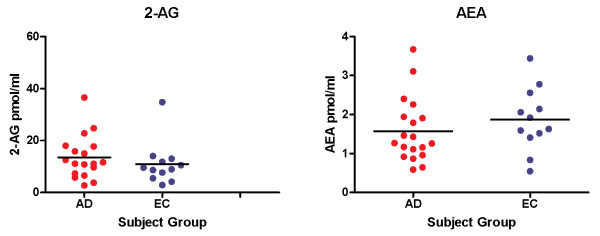
**Plasma concentrations of 2-AG and AEA in Alzheimer's disease subjects (AD) and elderly controls (EC)**.

**Figure 2:**
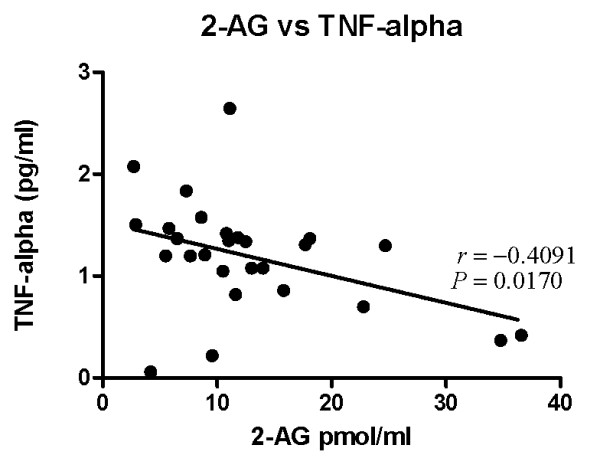
**Correlation of plasma 2-AG concentrations and TNF-α concentrations in pooled samples of AD subjects and elderly controls**.

Concentrations of AEA and 2-AG were successfully quantified in all plasma samples assayed. There were no significant differences in mean plasma concentrations of either AEA or 2-AG between AD subjects (N = 19, mean age 77.3, 53% female) and elderly controls (N = 12, mean age 73.4, 58% female) (AD:: AEA: 1.58 pmol/ml+/- 0.82; 2-AG:13.5 pmol/ml +/- 8.4; controls::AEA: 1.87 pmol/ml+/-0.87; 2-AG: 11.0 pmol/ml +/- 8.2)(Figure 1). There was no significant correlation between either AEA or 2-AG and C-reactive protein concentrations. There was a significant inverse correlation between 2-AG (but not AEA) concentrations and TNF-α concentrations (Pearson r = -0.41, p < 0.02) (Figure 2).

In CSF, 2-AG was successfully quantified in 35 subjects (mean 2-AG = 0.205 pmol/ml +/- 0.116; range 0.027–0.714). AEA was not detected in any CSF samples. The mean age of subjects was 62.7+/-8.0 yrs, and 71% were female. There was no significant correlation found between CSF 2-AG concentrations and any measured domain of cognition.

## Conclusion

In a sample of 19 subjects with AD and 12 elderly controls, we found no significant differences in concentrations of plasma eCBs. An argument could be made that this study was not sufficiently powered to detect difference. However, significant alterations in concentrations of plasma eCBs have been demonstrated in similarly sized samples of patients with other neuropsychiatric conditions. In a sample of 12 women with bulimia nervosa, 15 women with anorexia nervosa, and 15 healthy women, a significant increase in plasma AEA was reported in both bulimic and anorexic patients compared with controls; leptin concentrations were significantly inversely correlated with AEA concentrations in both anorexic patients and healthy controls [18]. Plasma concentrations of AEA were found to be significantly increased in schizophrenic patients in a sample of 12 patients with schizophrenia and 20 healthy controls [12].

Although a larger study would be necessary to conclusively dismiss any impact of AD pathology on circulating eCBs, our aim was to evaluate the utility of eCBs as diagnostic biomarkers for AD. There was sufficient power in the present study to detect a clinically useful discrepancy in eCB plasma concentrations had one existed in the populations represented by these samples. While this study does not support the utility of eCBs as diagnostic biomarkers of disease state in AD, longitudinal studies are required to evaluate whether perturbations in eCBs occur over the course of disease, and whether they are associated with particular presentations or rates of disease progression.

Elevations in inflammatory plasma proteins, most notably C-reactive protein, have been found to be associated cross-sectionally and prospectively with AD [27]. For this reason we chose to investigate the relationship between eCBs (as a potential biomarker for AD related inflammation) and C-reactive protein. Of note, in our plasma AD sample we did not find any significant differences in levels of C-reactive protein between AD patients and controls (data not shown). The relationship between eCBs and C-reactive protein might be investigated in samples with elevated levels of C-reactive protein, as there may be correlations in situations of systemic immune response. In order to elucidate the relationship between circulating eCBs and cytokines, we chose to focus on TNF-α as the preponderance of *in vitro *data seems to support the notion that eCBs exert some control over its production via the CB_2 _receptor. For instance, 2-AG inhibits the production of TNF-α production in both lipopolysacharide-stimulated mouse macrophages [28] and lipopolysacharide-stimulated microglial cells [29]. Most studies suggest a suppressive role for 2-AG in immune response, and our observation of an inverse correlation between circulating 2-AG and TNF-α would support this contention. It is important to note that the levels of TNF-α detected in our samples were very low, (mean 1.19 pg/ml +/- 0.55). There is probably very little physiologic relevance to these concentrations of TNF-α; however, for AD, a condition of at least local inflammation, 2-AG may play a crucial role in modifying immune response via its effect on TNF-α production. A recent study of signaling proteins that predict clinical AD has identified TNF-α underproduction as a risk factor for AD [30]; although very speculative, it is conceivable that 2-AG may play some role in this. The impact of circulating plasma eCBs on other cytokines is unclear, as is the influence of parenchymal eCBs on cytokines in regions of inflammation in brain. Further *in vivo *research is required to clarify these relationships.

There is little doubt that the active cannabinoid constituent Δ^9^-THC impacts learning and memory. Consistent with this, CB_1 _receptor localization has revealed abundant expression in hippocampus, cerebral cortex, cerebellum, and basal ganglia [31]. In the human cortex, density of receptor expression is highest in temporal and frontal lobes, and asymmetric, with increased receptor expression in the left hemisphere suggesting a relationship with verbal language and memory systems [32]. CB_1 _receptors in hippocampus and neocortex are distinctly expressed by GABAergic interneurons, and interact with endocannabinoids produced in post-synaptic neurons in a retrograde manner, with resultant depolarization-induced suppression of inhibition [33]. As modulation of inhibition generally has effects on long-term potentiation at excitatory synapses, and as the hippocampus plays such a crucial role in the anatomy of memory, the endocannabinoid system has become a focus of research interest in cognitive neuroscience. As adumbrated above, the direction of the relationship between eCBs and cognition remains unclear. Our failure to find an impact of CSF 2-AG concentrations on normative cognition does not necessarily mean that levels of eCBs in brain do not impact cognitive performance; what is seen in the CSF may not reflect what is present in hippocampus. In plasma, the mean circulating levels of 2-AG in our AD and controls samples were nearly 60 times those detected in the CSF of subjects undergoing cognitive testing described above (where AEA was not detected at all), most likely representing a greater proliferation of immune competent cells producing eCBs in plasma. eCBs may also exert an influence over cognition only in the context of dramatic overproduction, as may be the case in neuroinflammatory diseases such as AD. Additionally, our cognitive tests were somewhat circumscribed and other specific tests might be more sensitive at detecting cannabinoid-influenced performance. Future studies will be required to answer these questions.

## Conclusion

In summary, we did not find evidence suggesting a direct relationship between the eCB system and AD. The potential value of eCB quantification for AD and the functional relevance of eCBS as mediators of cognitive performance remains unclear. The neuropathological, animal, and cell culture reports which have implicated the eCB system in AD require focused translational experiments in clinical research to determine what consequence, if any, eCB functional variability has on cognition, disease development or progression.

## Competing interests

PD is a paid consultant to, and equity owner in, Applied Neurosolutions, Inc. The authors have no competing interests.

## Authors' contributions

JK designed study, wrote protocol, recruited subjects. MG performed lumbar puncture and helped with manuscript preparation. TG designed cognitive testing strategy, performed statistical analysis. PM and HK performed immunoassays (ELISA). HB, MP, and JMW designed and carried out eCB analysis in plasma and CSF. EC and PD aided in the design of the study, interpretation of data, and helped to draft the manuscript.
